# Effect of co-exposure to nickel and particulate matter on insulin resistance and mitochondrial dysfunction in a mouse model

**DOI:** 10.1186/1743-8977-9-40

**Published:** 2012-11-05

**Authors:** Xiaohua Xu, Xiaoquan Rao, Tse-Yao Wang, Silis Y Jiang, Zhekang Ying, Cuiqing Liu, Aixia Wang, Mianhua Zhong, Jeffrey A Deiuliis, Andrei Maiseyeu, Sanjay Rajagopalan, Morton Lippmann, Lung-Chi Chen, Qinghua Sun

**Affiliations:** 1 Davis Heart and Lung Research Institute, College of Medicine, The Ohio State University, Columbus, Ohio; 2 Division of Environmental Health Sciences, College of Public Health, The Ohio State University, Columbus, Ohio; 3 Department of Physiology, Hangzhou Normal University, Hangzhou, China; 4 Department of Environmental Medicine, New York University School of Medicine, Tuxedo, New York; 5 Division of Cardiology, College of Medicine, The Ohio State University, Columbus, Ohio

**Keywords:** Nickel, Inflammation, Insulin resistance, Air pollution

## Abstract

**Background:**

It has been well recognized that toxicity of fine ambient air particulate matter (PM_2.5_) may depend on its chemical constituents, including components such as soluble metals that may theoretically exert distinctive effects. We have recently demonstrated an important effect of PM_2.5_ on metabolic function. Since transition metals, such as nickel (Ni), represent an important component of exposure in certain environments, and may significantly influence the toxicity of inhalational exposure, we investigated the effects of Ni as a variable component of ambient PM_2.5_ exposure.

**Methods:**

Male ApoE knockout mice were exposed to filtered air (FA), fine-sized nickel sulfate particles alone (Ni) at 0.44 *μ*g/m^3^, concentrated ambient air PM_2.5_ (CAPs) at a mean of 70 *μ*g/m^3^, or CAPs+Ni in Tuxedo, NY, 6 hours/day, 5 days/week, for 3 months.

**Results:**

Exposure to Ni, irrespective of co-exposure to CAPs, resulted in body weight gain, while exposure to CAPs+Ni significantly enhanced fasting glucose and worsened insulin resistance measures (HOMA-IR), when compared with exposure to CAPs alone. CAPs+Ni exposure induced a significant decrease in phosphorylation of AMP-activated protein kinase (AMPK) α. Exposure to Ni or CAPs+Ni significantly induced microcirculatory dysfunction and increased monocytic cell infiltration into lung and adipose, and decreased uncoupling protein 1 expression at gene and protein levels and several brown adipocyte-specific genes in adipose tissue.

**Conclusions:**

Ni exposure has effects on metabolic and inflammatory parameters that are comparable to that of CAPs. Additionally, Ni synergistically exacerbates CAPs-induced adverse effects on some of, but not all of, these parameters, that may be mediated via the AMPK signaling pathway. These findings have important implications for inhaled transition metal toxicity that may exert synergistic effects with other PM_2.5_ components.

## Background

Exposure to levels of fine ambient particulate matter (PM_2.5_, particles less than 2.5 *μ*m in aerodynamic diameter), even at levels below the current annual average National Ambient Air Quality Standard (15 *μ*g/m^3^), have been associated with excess annual mortality and morbidity [1]. However, the components of PM_2.5_ responsible for these effects continue to remain unclear.

It has, however, been well recognized that toxicity of the PM_2.5_ depends, at least in part, on the specific chemicals that are present, and that metals are often implicated as causative agents. Nickel (Ni), a transition metal in the earth’s crust, is found at highly variable concentrations in PM_2.5_ air pollution [2-4]. A number of epidemiologic studies have indicated significant correlations between respiratory cancers and occupational exposures to Ni [5-7]. Also, Laden *et al.*[8] demonstrated that Ni was positively associated with daily deaths through studies in six U.S. Cities, and Lippmann *et al.*[1] showed significant associations of Ni with daily deaths in 60 U.S, cities, as well as changes in heart rate variability (HRV) in mice that were highly correlated with Ni levels in the air that they inhaled. Back trajectory analyses for the highest Ni concentrations led to the vicinity of the largest nickel smelter in North America at Sudbury, Ontario.

We have previously demonstrated that PM_2.5_ air pollution potentiates multiple facets of metabolic function including inflammation, adiposity, and brown adipose dysfunction, and that it is a factor that contributes to the development of insulin resistance (IR) through these various pathways [9-14]. Nevertheless, little is known about the effect of Ni when combined with other PM_2.5_ components. In order to gain a better understanding, the current study focused on changes in inflammation, mitochondrial function, and metabolic parameters after individual or mixed exposure to PM_2.5_ and/or added Ni. We hypothesized that co-exposure to PM_2.5_ and Ni might exert some synergistic effects on IR and metabolic disorders.

## Materials and methods

### Animals

Male Apolipoprotein E deficient (ApoE^−/−^) mice (8-week-old) were purchased from Jackson Laboratories (Bar Harbor, MA). All mice were given 7 days to adjust to their new environment, and then randomly assigned to 4 groups: FA (exposed to filtered air), CAPs (inertially concentrated PM_2.5_), Ni (fine-sized NiSO_4_), and CAPs+Ni. All groups were fed rodent standard laboratory chow and allowed to eat *ad libitum* throughout the duration of the study. The mice were housed on a 12-hour light–dark cycle in a temperature-controlled room at 25°C. NIH guidelines for the care and use of laboratory animals were strictly followed, and all experiments were approved by the Animal Care and Use Committee at The Ohio State University (Protocol Number: 2008A0006-R1) and the New York University School of Medicine (Protocol Number: 100805–02).

### Whole-body inhalational exposure protocol

As shown in Additional file 1: Figure S1, the mice were exposed to the northeastern regional background CAPs, produced using a modified versatile aerosol concentration enrichment system, for 6 hours/day, 5 days/week between Sep. 8 and Dec. 17, 2009, at the AJ Lanza Laboratory in the Department of Environmental Medicine of New York University School of Medicine in Sterling Forest (Tuxedo, NY), as described previously [9,10,15]. There was a 9-fold concentration factor for the CAPs.

The mice were exposed to Ni (NiSO_4_, produced using a Collison nebulizer [BGI, Waltham, MA]) at a nominal concentration of 440 *n*g/m^3^, which was approximately 9-fold greater than that seen in New York City in the winter [16]. The exposures lasted for 6 hours/day, one day a week (on Wednesdays) between Sep. 8 and Nov. 2, 2009 using the same whole-body exposure system as that used in the CAPs exposure. Because we did not see acute changes in heart rate and heart rate variability by ECG transmitters with Wednesdays only protocol, we switched from Wednesdays only exposure to 5 days/week exposure until the end of the study (between Nov. 3 and Dec. 17, 2009). On non-Ni exposure days between Sep. 8 and Nov. 2, 2009, these mice were exposed to filtered air (FA). The mice assigned to CAPs+Ni were exposed to CAPs (6 hours/day, 5 days/week) but with NiSO_4_ added to the CAPs stream on the same day as Ni alone group (see above). The mice in the FA group were exposed to an identical protocol, with the exception that a high efficiency particulate air (HEPA) filter was positioned in the inlet valve to the exposure system to remove all of the PM_2.5_ from that air stream, as detailed previously [10,15]. CAPs and Ni mass concentrations were determined gravimetrically [10,15]. X-ray fluorescence (XRF) spectroscopy (ES6600, Jordan Valley) was used to determine the concentrations of selected elements in the CAPs and Ni exposures. Particle size distributions of CAPs and Ni were determined using a SMPS system (TSI, Minneapolis, MN).

### Blood glucose homeostasis and insulin resistance (IR) assessment

After the 3 months of the various exposures summarized in Table 1, the mice were fasted overnight and dextrose (2 mg/g body weight, Hospira, Lake Forest, IL) was injected intraperitoneally. Blood glucose measurements were conducted with an Elite Glucometer (Bayer) every 30 minutes for 2 hours after the dextrose injection. Insulin levels were determined using an Ultra Sensitive Mouse Insulin ELISA Kit (Crystal Chem Inc., Downers Grove, IL). IR was estimated using the homeostasis model assessment method (HOMA) based on 1 mg of insulin as equivalent to 24 IU, using the formula HOMA-IR = [fasting insulin concentration (ng/ml) × 24 × fasting glucose concentration (mg/dl)]/405 [13].

**Table 1:** **Mean daily PM**_**2.5**_**mass concentrations (μg/m**^**3**^**) and Ni concentrations (ng/m**^**3**^**) of the exposure atmospheres**

	**Ambient PM**_**2.5**_	**FA**	**CAPs**	**Ni**	**CAPs+Ni**
PM_2.5_ (*μ*g/m^3^)	7.4 ± 4.4	ND	69.6 ± 48.4	ND	66.5 ± 44.6
Ni (ng/m^3^)	ND	ND	0.9 ± 5.5	440.6 ± 557.3	467.9 ± 601.1

Data are expressed as mean ± standard deviation. FA: filtered air. ND: not detectable.

### Measurement of serum cytokines

After the end of the exposures, peripheral blood was collected, spun, and serum was stored at −80°C for the analysis of cytokines. Cytokine levels were determined by Cytometric Bead Array (BD Biosciences, San Jose, CA) according to the manufacturer’s instructions. Serum was incubated with beads specific for tumor necrosis factor (TNF), interferon γ (IFN-γ), monocyte chemoattractant protein 1 (MCP-1), interleukin 6 (IL-6), IL-10 and IL-12p70. The total amount of cytokines was then determined using a BD LSR II instrument and analyzed by the BD CBA software (BD Biosciences).

### Intravital microscopy

After the exposures, the intravital microscopy was performed as described previously [9]. Briefly, after the mice were anesthetized intraperitoneally by a mixture of ketamine (100 mg/kg) and xylazine (20 mg/kg), the cremaster muscle was exteriorized, mounted, and superfused with pre-warmed Ringer’s lactate (37°C). Leukocyte-endothelial cell interactions were obtained using a Nikon Eclipse FN1 microscope (Nikon, Tokyo, Japan) with a 40×/0.80-W water-immersed objective and Metamorph software (version 7.1.2.0, Metamorph, Downingtown, PA). The numbers of rolling and adherent cells in cremasteric muscle were determined in a 100-*μ*m vessel length per 30 seconds per image field (1.57 × 10^5^*μ*m^2^), and the data presented were averaged from 10–15 venules per mouse. Cells that remained stationary for at least 5 seconds were considered “adherent” cells [9].

### Immunohistochemistry

Immunohistochemical staining was performed as described elsewhere [17], with some modification. Briefly, deparaffinized sections (5 *μ*m) of lung, epididymal white adipose tissue (eWAT), and interscapular brown adipose tissue (iBAT) were subjected to heat-induced antigen retrieval by incubation in Retrieve-all-1 unmasking solution (Signet Labs, Dedham, MA), followed by washing with phosphate buffered saline (PBS). The slides were treated with 0.3% H_2_O_2_ and incubated with primary antibody overnight at 4°C, followed by appropriate horseradish peroxidase (HRP)-conjugated secondary antibody. The staining was developed using Fast 3,3’-diaminobenzidine tablet sets (D4293; Sigma, St. Louis, MO), while the sections were counterstained with hematoxylin and examined by light microscopy. Cryostat thoracic aorta sections (8 *μ*m) were stained by the same procedures without the heat-induced antigen retrieval. The primary antibodies were rat anti-mouse F4/80 (AbD Serotec, Raleigh, NC) and rat anti-mouse UCP1 (Abcam Inc., Cambridge, MA). All measurements were conducted in a double-blinded manner by two independent investigators.

### Dihydroethidium (DHE) staining

The oxidative fluorescent probe DHE was used to evaluate *in situ* superoxide (O_2_^-^) production on cryosections of iBAT. DHE staining was performed as described previously [12].

### Transmission electron microscopy (TEM)

To investigate changes in mitochondrial size and number *in situ* between groups, we examined the ultrastructure of eWAT by TEM, as described elsewhere [18].

### Real-time PCR

The eWAT and iBAT from the mice were excised, minced, and RNA was isolated using TRIzol Reagent (Invitrogen) according to the manufacturer’s instructions. Total RNA levels were then converted into cDNA using the High Capacity cDNA Reverse Transcription Kit (Applied biosystems, Foster City, CA). The quantification of gene expression was determined by real-time PCR. All reactions were performed under the same conditions: 50°C for 2 minutes, 95°C for 10 minutes, 40 cycles of 95°C for 15 seconds, and 60°C for 1 minute. The primers for mouse *Ucp1*, PRD1-BF1-RIZ1 homologous domain containing 16 (*Prdm16*), peroxisome proliferator-activated receptor-γ coactivator 1-α (*Pgc-1α*), type 2 iodothyronine deiondinase (*Dio2*), cell death-inducing DNA fragmentation factor, alpha subunit-like effector A (*Cidea*), elongation of very long chain fatty acid 3 (*Elovl3*), and *β–actin* are showed in Additional file 2: Table S1. *Beta–actin* was used as the control gene and all data are represented as relative mRNA expression on gene expression.

### Western blotting

Twenty micrograms of protein from iBAT was separated by sodium dodecyl sulfate-polyacrylamide gel, and then transferred to PVDF membranes (Bio-Rad, Hercules, CA). Membranes were incubated with primary antibody against UCP1 (Abcam), AMP-activated protein kinase α (AMPKα) and phospho-AMPKα (Thr172, Cell Signaling) overnight at 4°C, respectively. Membranes were then washed and incubated with HRP-conjugated secondary antibody. Protein bands were visualized by enhanced chemiluminescence (Amersham, Little Chalfont, Buckinghamshire, UK).

### Statistical analysis

Data are expressed as mean ± s.e. unless otherwise indicated. The results of experiments were analyzed by two-way analysis of variance (ANOVA), and were performed using Graphpad Prism v5.0 (GraphPad Software, San Diego, CA). In all cases a *P* value of < 0.05 was considered statistically significant.

## Results

### Exposure characterization

As shown in Table 1, the ambient mean daily PM_2.5_ mass concentration at the study site was 7.4 ± 4.4 *μ*g/m^3^, while the mean concentrations of CAPs and CAPs+Ni in the exposure chamber was 69.6 ± 48.4 and 66.5 ± 44.6 *μ*g/m^3^, respectively (≈9-fold concentration from the ambient levels). The mean concentrations of Ni in these exposure atmospheres were 0.9 ± 5.5, 440.6 ± 557.3 and 467.9 ± 601.1 ng/m^3^ in the CAPs, Ni and CAPs+Ni groups, respectively. The elemental concentrations, as measured by ED-XRF analysis, are presented in Additional file 2: Table S2.

### Physical characteristics

Table 2 illustrates the characteristics of the animals exposed to CAPs, Ni, and CAPs+Ni for 3 months. At baseline there were no significant differences in total body weight among the groups (data not shown). After 3 months of exposure to CAPs, Ni, or CAPs+Ni, the mice exposed to Ni significantly had gained weight with or without CAPs treatment, whereas CAPs alone did not induce significant changes in total body weight. Mice exposed to CAPs alone, but not Ni, showed a significant increase in the weights of eWAT, although we did not observe any significant differences in iBAT, heart weight, or ratio of heart weight to body weight.

**Table 2:** Characteristics of the mice in response to the exposures

	**FA**	**CAPs**	**Ni**	**CAPs-Ni**
Body weight, g	31.4 ± 0.5	30.1 ± 1.0	33.1 ± 0.4^*#^	32.7 ± 0.2^*#^
eWAT weight, g	0.26 ± 0.02	0.32 ± 0.01^*^	0.28 ± 0.08	0.26 ± 0.03
iBAT weight, g	0.06 ± 0.01	0.06 ± 0.01	0.05 ± 0.01	0.06 ± 0.01
Heart weight, g	0.15 ± 0.01	0.15 ± 0.01	0.15 ± 0.01	0.14 ± 0.01
Heart weight/BW, g/kg	4.85 ± 0.3	4.66 ± 0.38	4.53 ± 0.24	4.13 ± 0.14

Values are means ± s.e. N = 5–6. ^*^P < 0.05 *vs* FA; ^#^*P* < 0.05 vs CAPs. BW, body weight.

### Glucose intolerance and IR

**Figure 1:**
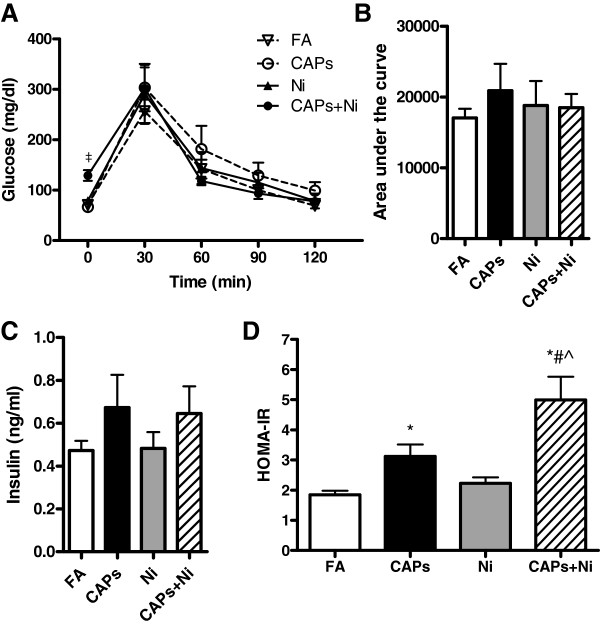
**Effect of the exposure to CAPs, Ni, or CAPs+Ni on glucose homeostasis.A**, Intraperitoneal glucose tolerance test (IPGTT). **B**, The glucose area under the curve calculated from the glucose tolerance test from (A). **C**, Fasting insulin level. **D**, The homeostasis model assessment of insulin resistance (HOMA-IR) index of insulin sensitivity. N = 5–6. ^‡^*P* < 0.001 *vs.* FA, CAPs, or Ni group at each time; ^*^P < 0.05 *vs.* FA; ^#^*P* < 0.05 *vs.* CAPs, ^^^*P* < 0.05 *vs.* Ni group. FA, filtered air; CAPs, concentrated ambient particles (particulate matter, less than 2.5 *μ*m in diameter); Ni, nickel.

Figure 1 shows the metabolic parameters after exposure to CAPs, Ni, and CAPs+Ni. Exposure to CAPs+Ni significantly increased fasting glucose levels, although we did not find any significant differences in glucose tolerance profile among all the groups (Figure 1A,B). There were no significant differences in the fasting insulin levels (Figure 1C). Nevertheless, HOMA-IR index was increased with CAPs exposure and was the highest with co-exposure of CAPs with NiSO_4_ (Figure 1D).

### Changes in serum cytokines

**Figure 2:**
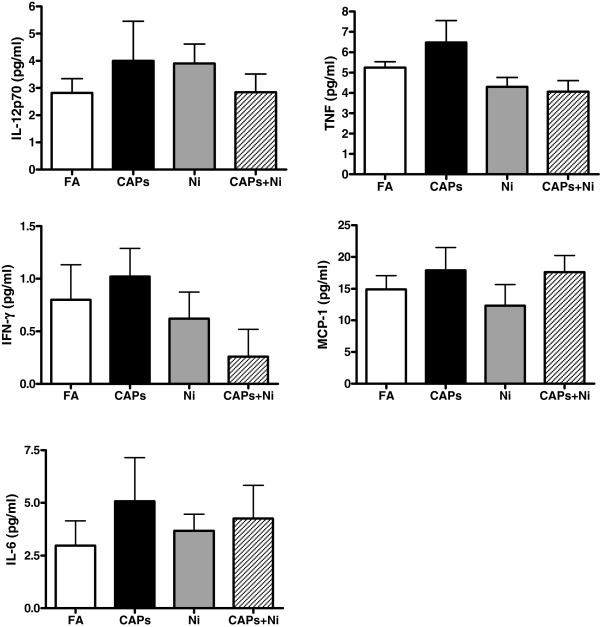
**The levels of circulating inflammatory cytokines (IL-12p70, TNF, IFN-γ, MCP-1, and IL-6) in response to CAPs, Ni, or CAPs+Ni exposure.** N = 5–6.

Figure 2 shows systemic inflammatory cytokine levels after exposure to CAPs, Ni, and CAPs+Ni for 3 months. There were no significant differences in the levels of IL-12p70, TNF, IFN-γ, MCP-1, or IL-6 among all these groups, and the levels of IL-10 were undetectable.

### Exposure to CAPs and NiSO_4_ elicited increased monocytic cell infiltration and superoxide production, and decreased UCP1 expression

**Figure 3:**
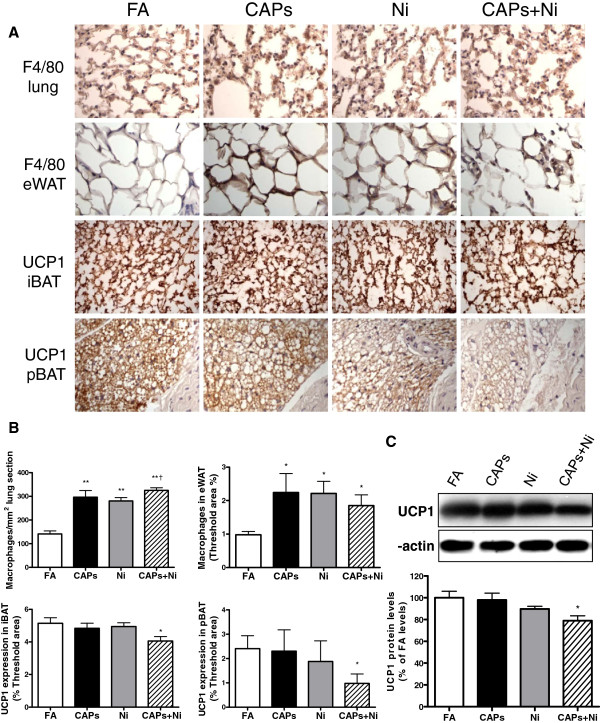
**Exposure to CAPs, Ni, or CAPs+Ni induces increased monocytic cell infiltration and reduces UCP1 expression.A** and **B**, Representative images **(A)** and statistical analysis **(B)** of immunohistochemistry for F4/80^+^ macrophages in the lung and eWAT, and UCP1 expression in the pBAT and iBAT depots. Original magnification, ×200. N = 5–6. ^*^P < 0.05 *vs.* FA; ^**^P < 0.001 *vs.* FA; ^†^*P* < 0.05 *vs.* Ni group. **C**, Western blotting for UCP1 expression in interscapular adipose tissue. Upper panel shows the representative western blotting bands, and lower panel is the statistical analysis. N = 5–6. ^*^P < 0.05 *vs.* FA group. iBAT, interscapular brown adipose tissue; pBAT, perivascular brown adipose tissue.

**Figure 4:**
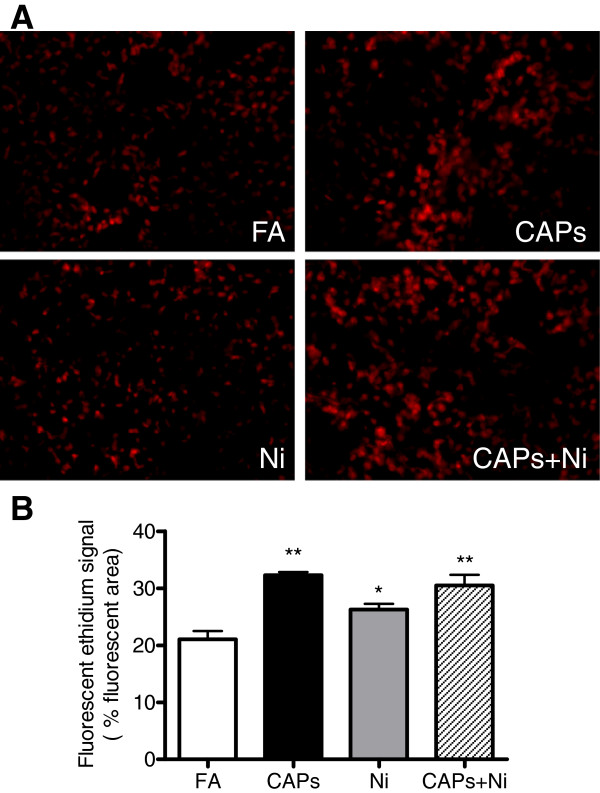
**Effect of exposure to CAPs, Ni, or CAPs+Ni on superoxide production in iBAT depots.A** and **B**, Representative images **(A)** and quantification **(B)** for superoxide production, as determined by dihydroethidium (DHE) staining. Original magnification × 200. N = 5–6. ^*^P < 0.05 *vs.* FA group. ^**^P < 0.001 *vs.* FA group.

As shown in Figure 3A-B, exposure to CAPs, Ni, and CAPs+Ni significantly induced an increase in the monocytic cell (F4/80^+^) infiltration into the lung and eWAT when compared to the FA group. UCP1 expression was significantly reduced in iBAT and perivascular brown adipose tissue (pBAT) depots by exposure to the CAPs+Ni. The decrease in UCP1 expression was further confirmed by Western blotting measurement in iBAT depots, as shown in Figure 3C. Additionally, as shown in Figure 4A-B, exposure to either CAPs or Ni alone, or combination of both significantly induced superoxide production in iBAT as determined by DHE staining.

### Exposure to CAPs and NiSO_4_ induced microcirculatory dysfunction

**Figure 5:**
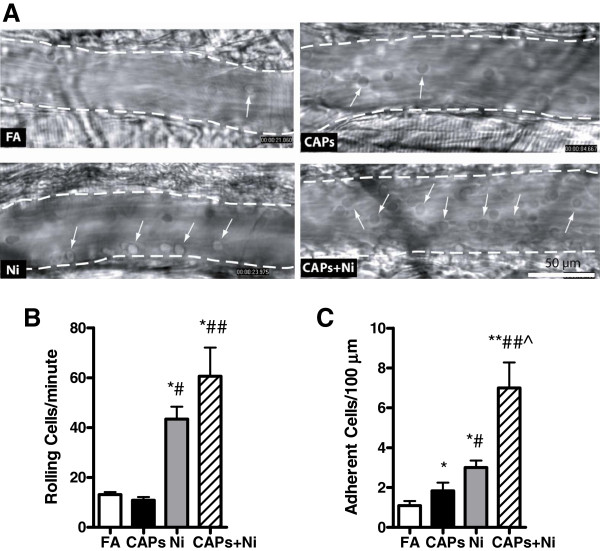
**Exposure to CAPs, Ni, or CAPs+Ni induces microvascular dysfunction.A**. Representative images of adherent leukocytes in the cremasteric microcirculation (cells with arrow heads indicate adherent monocytes, cells without arrow heads indicate rolling leukocytes) by intravital microscopy. Scale bar, 50 μm. **B** and **C**, Statistical analysis of monocyte rolling flux **(B)** and adhesion **(C)** in cremasteric microcirculation. N = 5–6. ^*^P < 0.05 *vs.* FA; ^**^P < 0.001 *vs.* FA; ^#^*P* < 0.05 *vs.* CAPs; ^##^*P* < 0.001 *vs.* CAPs; ^^^*P* < 0.05 *vs.* Ni group.

At the end of the exposure to CAPs and/or NiSO_4_, intravital microscopy was performed to evaluate the number of the rolling and adherent cells, as an index of recruitment into the tissue depots. As shown in Figure 5A-C, CAPs exposure resulted in a significant increase in adherent monocytes in the microcirculation when compared to the FA-exposed mice. Also, Ni exposure led to a significant increase in adherent and rolling monocytes in the microcirculation when compared to the FA- and CAPs-exposed mice. Moreover, there was an important synergistic effect of Ni in terms of exaggerating the CAPs effects. These results indicate that both CAPs and NiSO_4_ induce microcirculatory dysfunction, while the combination of CAPs and NiSO_4_ had synergistic effects, and may have augmented this dysfunction.

### Exposure to CAPs and NiSO_4_ led to in-situ mitochondrial changes

**Figure 6:**
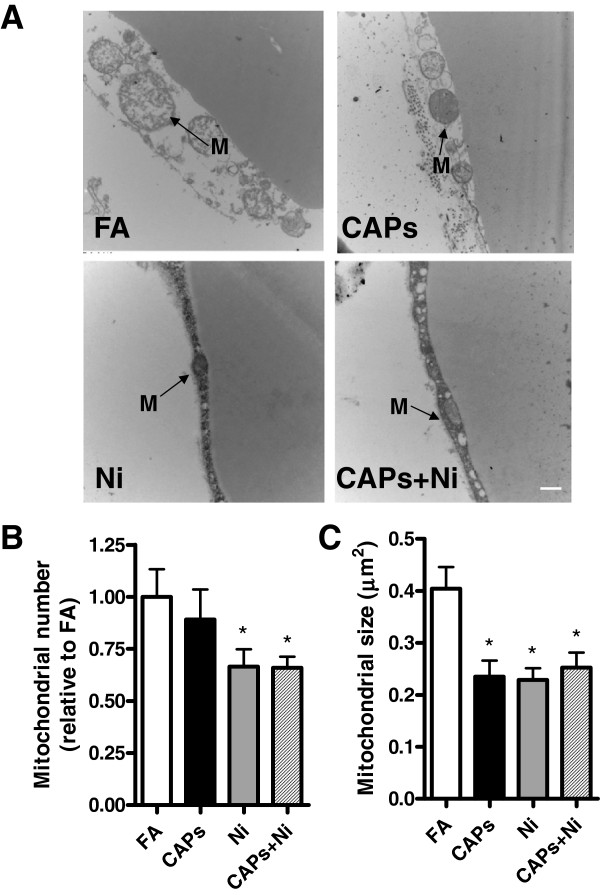
**Effect of exposure to CAPs, Ni, or CAPs+Ni on mitochondria in visceral adipose tissue by transmission electronic microscopy (TEM).** Representative TEM images (**A**) and quantification of mitochondrial number (**B**) and size (**C**). Original magnification, ×18,500. Scale bar, 500 nm. Arrows indicate the position of mitochondria (M). ^*^P < 0.05 *vs.* FA group.

Figure 6 shows the changes in mitochondria in eWAT in response to CAPs and/or NiSO_4_ exposure. Two quantitative parameters were used to assess mitochondria: mitochondrial number (Figure 6B) and size (Figure 6C). NiSO_4_, with or without CAPs exposure, significantly decreased mitochondrial number, while CAPs and/or NiSO_4_ exposure resulted in reduced size of mitochondria in the white adipose depots.

### Exposure to CAPs and NiSO_4_ affected brown adipocyte-specific gene profiling

**Figure 7:**
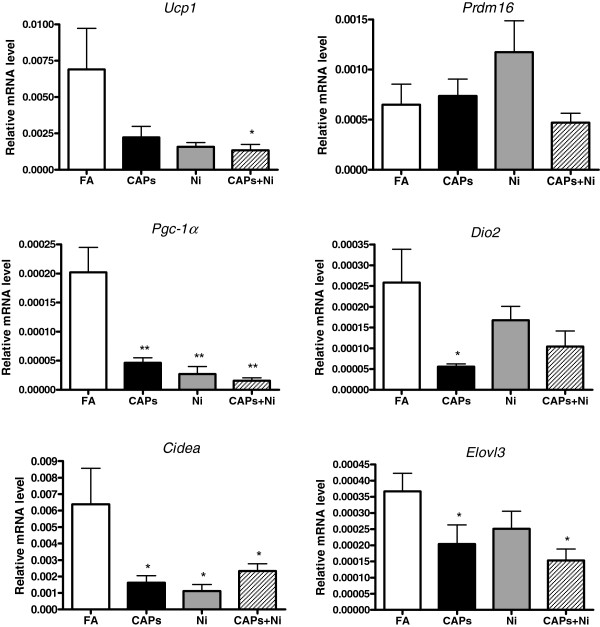
**Alteration of brown adipocyte-specific gene expression in eWAT in response to CAPs, Ni, or CAPs+Ni exposure.** N = 5–6. ^*^P < 0.05 *vs.* FA group; ^**^P < 0.001 *vs.* FA group. Uncoupling protein 1 (*Ucp1*), PRD1-BF1-RIZ1 homologous domain containing 16 (*Prdm16*), peroxisome proliferator-activated receptor-γ coactivator 1-α (*Pgc-1α*), type 2 iodothyronine deiondinase (*Dio2*), cell death-inducing DNA fragmentation factor, alpha subunit-like effector A (*Cidea*), elongation of very long chain fatty acid 3 (*Elovl3*).

**Figure 8:**
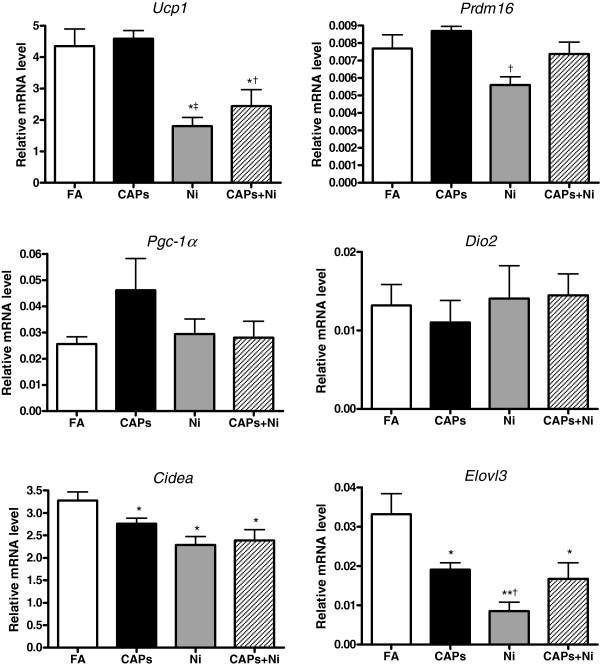
**Alteration of brown adipocyte-specific gene expression in iBAT in response to CAPs, Ni, or CAPs+Ni exposure.** N = 5–6. ^*^P < 0.05 *vs.* FA group; ^**^P < 0.001 *vs.* FA group; ^†^*P* < 0.05 *vs.* CAPs group; ^‡^*P* < 0.001 *vs.* CAPs group. Uncoupling protein 1 (*Ucp1*), PRD1-BF1-RIZ1 homologous domain containing 16 (*Prdm16*), peroxisome proliferator-activated receptor-γ coactivator 1-α (*Pgc-1α*), type 2 iodothyronine deiondinase (*Dio2*), cell death-inducing DNA fragmentation factor, alpha subunit-like effector A (*Cidea*), elongation of very long chain fatty acid 3 (*Elovl3*).

To determine gene expression changes in response to CAPs and/or NiSO_4_ exposure, we measured the expression levels of the brown adipocyte-specific gene profiles by real-time PCR analysis in eWAT (Figure 7). We observed that the mRNA levels of the brown adipocyte-specific gene *Ucp1* and *Pgc-1α* decreased in response to NiSO_4_ co-exposure with CAPs. The mRNA levels of *Dio2* were significantly reduced by CAPs exposure compared to the FA-exposed mice. In addition, *Cidea* and *Elovl3* gene expressions were significantly lowered by CAPs and/or NiSO_4_ exposure, although there were no significant differences in *Prdm16* gene expression among the groups. To evaluate if CAPs and/or NiSO_4_ exposure might change the brown adipocyte-specific gene profiles in BAT, we measured these gene expressions in iBAT. As shown in Figure 8, the mRNA levels of *Ucp1*, *Cidea*, and *Elovl3* were significantly decreased by NiSO_4_, with or without CAPs exposure, while CAPs exposure also reduced the mRNA levels of *Cidea* and *Elovl3* when compared to the FA-exposed mice.

### Exposure to CAPs and NiSO_4_ suppressed AMP-activated protein kinase (AMPK) phosphorylation

**Figure 9:**
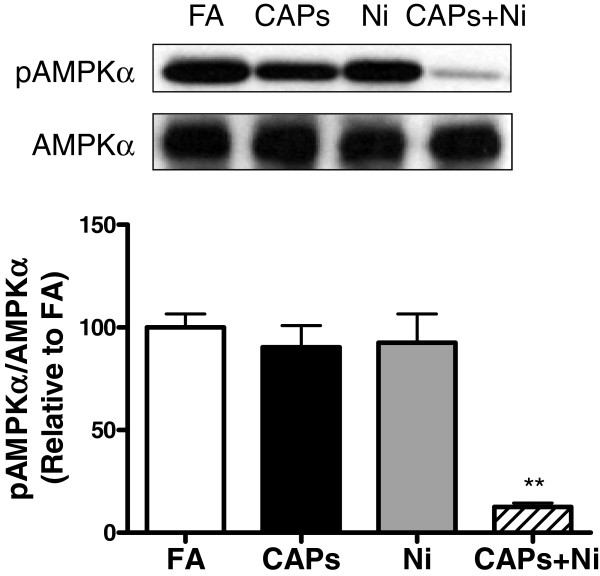
**Western blotting for the evaluation of phosphorylated and total AMPKα expression in the liver.** Upper panel shows the representative western blotting bands, and lower panel is the statistical analysis. N = 5–6. ^**^P < 0.0001 *vs.* FA, CAPs, or Ni group.

AMPK is a molecule that has been shown to be important in lipid metabolism. As shown in Figure 9, simultaneous exposure to CAPs and NiSO_4_ significantly inhibited the phosphorylation of AMPK in the liver, indicating that AMPK signaling pathway might be involved in the effect of CAPs and NiSO_4_ exposure on mitochondrial dysfunction.

## Discussion

Our work provides preliminary evidence for significant interactions between a metal ion (Ni) and CAPs in terms of metabolic function. There are several important findings in this study. First, exposure to the combination of CAPs and Ni resulted in an increase in fasting glucose levels and a higher HOMA-IR index than exposure to CAPs alone. These changes were associated with decreased AMPK activity in the liver. Second, Ni exposure was equipotent to CAPs in terms of inflammatory cell infiltration in peripheral tissues, including the lung. Third, Ni co-exposure with CAPs synergized an induction of significant leukocyte adhesion and microcirculatory dysfunction. Finally, exposure to CAPs and Ni induced changes that are highly consistent with mitochondrial dysfunction.

Air pollution is a heterogeneous and complex mixture of compounds in gases, liquid droplets and solid particulate matter. Thus, the heterogeneous composition of the droplets and solid particles indicates that PM exposure may contribute to cellular and molecular toxicity through various pathways. Particle size, surface area, and chemical composition influence the health risk posed by PM. Exposure to ambient PM air pollution is associated with increased mortality and morbidity in susceptible populations. A number of studies have revealed that exposure to CAPs is related to acute and chronic effects on cardiac function, increased amounts of more invasive aortic plaque, inflammation, and insulin resistance, as well as adiposity [9,10,19-22]. It has been reported that soluble transition metals, such as iron (Fe), Ni, and vanadium (V), are responsible for the majority of residual oil fly ash toxicity [23,24]. Lippmann *et al.*[1] have demonstrated that Ni is significantly associated with acute changes in heart rate and its variability in CAPs-exposed and sham-exposed ApoE knockout mice. In this study, we used the whole body exposure system and exposed the same strain of the animal to CAPs and/or NiSO_4_ for 3 months. The concentration of Ni was expressed in terms of ng/m^3^, and it was much lower than the concentrations in any of the studies in which pure Ni compounds were used. We found that exposure to CAPs+Ni significantly induced inflammation in lung and adipose tissue, and enhanced the fasting glucose level and insulin resistance, although there was no significant difference in the intraperitoneal glucose tolerance test. In addition, exposure to NiSO_4_ exacerbated the microcirculatory dysfunction resulting from CAPs exposure. These findings suggest that Ni, together with the much larger mass concentration of the CAPs, has synergistic effects on these adverse health conditions.

Recent data in adult humans suggest an important link between BAT-mediated thermogenesis and obesity [25]. We have previously shown that long-term exposure to similar CAPs concentrations (for 10 months) induced a visible decrease in the iBAT and mitochondrial sizes [12]. These changes were accompanied by an increase in excess oxidative and nitrosative stress in BAT, coordinate with Phase II antioxidant gene induction including NF-E2-related factor 2 (Nrf2), NAD(P)H quinone oxidoreductase 1 (Nqo1) and glutamate-cysteine ligase modifier subunit (Gclm). BAT expressions of Ucp1 and Pgc-1α were decreased with CAPs exposure, while Prdm16, Pgc-1α, and Pparg2 were significantly decreased in the WAT, suggestive of downregulation of pathways that modulate insulin sensitivity in adipose [12]. Similar results were seen with the ApoE^−/−^ model used in this study [14]. UCP1, which is specifically expressed in BAT mitochondria, is largely responsible for the uncoupling of respiration from ATP synthesis, resulting in dissipation of energy as heat [26]. In addition to UCP1, proteins such as Dio2 and PGC-1α have also been shown to be highly enriched in BAT, but low in WAT [27,28]. Furthermore, PGC-1α has been shown to coordinate multiple physiological cues for mitochondrial biogenesis and activity [29]. In this study, we demonstrated that exposure to CAPs and Ni reduces the brown adipocyte-specific gene expression in the BAT as well as WAT, suggesting that exposure to CAPs and Ni may induce important alteration in BAT and/or BAT-like phenotypic changes in WAT.

AMPK activity, measured as Thr172 phosphorylation, was reduced in the metabolically active tissues of the mice exposed to CAPs+Ni. AMPK, an enzyme central to cellular bioenergetics, is considered a major metabolic regulator at both cellular and whole-body levels [30], and may regulate energy expenditure by modulating NAD^+^ metabolism and SIRT1 activity [31]. Activation of AMPK in the liver, skeletal muscle, and adipose tissue improves the status of type 2 diabetes [32]. AMP binds and activates AMPK, primarily by causing conformational changes that allow Thr172 phosphorylation to occur by upstream kinases. Yuan *et al.*[32] reported that second-hand smoke inhibits AMPK and ACC phosphorylation, suggesting AMPK is critically involved in the adverse effects of smoking.

Characterization of the effects of inhalation exposures, and zeroing in on the PM_2.5_ sources, is challenging for a variety of reasons. First of all, ambient PM_2.5_ is intrinsically complex, with thousands of chemical components. Second, studies of inhalation exposures that mimic the real world scenarios in a laboratory environment require technical sophistication. Third, the data previously generated by exposing animal models to single metals have been either largely negative or hard to relate to data generated in *in vitro* experiments. In addition, a “knockout” design involving the elimination of certain metals from ambient air for an inhalation exposure is technically infeasible. To address those issues, we proposed an “overexpression” design by adding Ni to CAPs in comparison with CAPs alone, Ni alone, or FA. This design not only enabled us to address the differences among those groups, especially in indicating if Ni played a significant role in the development of the disorders that have been associated with ambient air PM_2.5_ exposures, but also reflected the real situations in some regions/countries of the world where the Ni levels in the ambient air are relatively high [1,33,34].

In summary, our data suggest that Ni at a much lower concentration than that of CAPs (at a realistic level of CAPs exposure), can enhance metabolic disorders, mitochondrial dysfunction, and the monocytic cell infiltration into lung and adipose tissue. Therefore, they may explain an important role for co-exposure to Ni and CAPs in the development of metabolic disorders, and suggest an important public health impact of combined Ni and PM_2.5_ air pollution. Further understanding of the mechanism by which exposure to Ni and CAPs causes these adverse effects may provide novel prevention and therapeutic strategies for better control and treatment of metabolic disorders such as obesity, type 2 diabetes, and insulin resistance. The clinical implications of our results would be strengthened by further elucidation of the detailed mechanisms, and by alteration of the signaling pathways in exposed mice to assess possible improvement of mitochondrial biogenesis and reduction of the adverse health effects.

## Abbreviations

AMPK: AMP-activated protein kinase; BAT: Brown adipose tissue; CAPs: Concentrated fine particulate matter; Cidea: Cell death-inducing DNA fragmentation factor, alpha subunit-like effector A; Dio2: Type 2 iodothyronine deiondinase; Elovl3: Elongation of very long chain fatty acid 3; FA: Filtered air; HOMA: Homeostasis model assessment; HEPA: High efficiency particulate air; HRP: Horseradish peroxidase; HRV: Heart rate variability; IFN-γ: Interferon gamma; IL-6: Interleukin 6; IPGTT: Intraperitoneal glucose tolerance test; IR: Insulin resistance; MCP-1: Monocyte chemoattractant protein 1; Ni: Nickel; NiSO_4_: Nickel sulfate; Pgc-1α: Peroxisome proliferator-activated receptor-γ coactivator 1-α; PM_2.5_: Particulate matter less than 2.5 μm in aerodynamic diameter; Prdm16: PRD1-BF1-RIZ1 homologous domain containing 16; TNF-α: Tumor necrosis factor-α; UCP1: Uncoupling protein 1; XRF: X-ray fluorescence.

## Competing interests

The authors declare that they have no competing financial interests

## Authors' contributions

XX, XR, TYW, SYJ, ZY, CL, AW, MZ, JAD, and AM performed the experiments and contributed to acquisition of data. XX, XR, TYW, SYJ, ZY, CL, MZ, JAD, AM, and LCC analyzed the data and interpreted the results. MZ and LCC contributed to PM_2.5_ exposure of the animals. The manuscript was written by XX and revised critically by SR, ML, LCC, and QS. All authors read, corrected and approved the manuscript.

## Supplementary Material

Additional file 1 **Figure S1. Schematic diagram for the whole-body inhalational exposure protocol.** Click here for file

Additional file 2 **Table S1. Primers used for real-time PCR.Table S2. Selected elements in the exposure atmospheres measured using XRF.** Click here for file
