# Supplementary material for: Effect of co-exposure to nickel and particulate matter on insulin resistance and mitochondrial dysfunction in a mouse model
Source: Part Fibre Toxicol. 2012 Nov 5;9:40. doi: 10.1186/1743-8977-9-40 (PMC3545913; doi:10.1186/1743-8977-9-40)
Supplement: Additional file 1 — Figure S1. Schematic diagram for the whole-body inhalational exposure protocol. [file 1743-8977-9-40-S1.pptx]

## Slide 1
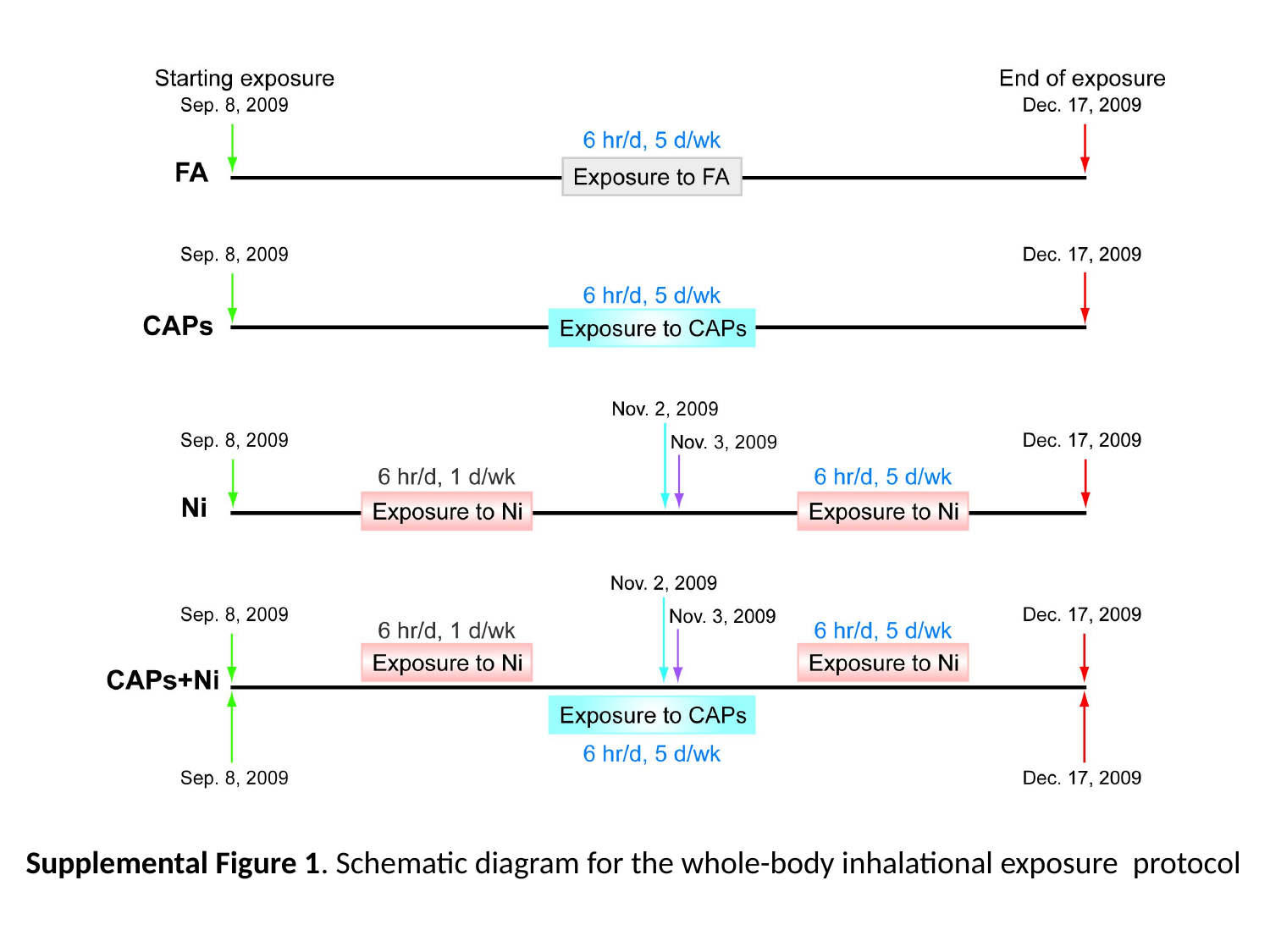

Supplemental Figure 1. Schematic diagram for the whole-body inhalational exposure protocol
