# Supplementary material for: Effect of co-exposure to nickel and particulate matter on insulin resistance and mitochondrial dysfunction in a mouse model
Source: Part Fibre Toxicol. 2012 Nov 5;9:40. doi: 10.1186/1743-8977-9-40 (PMC3545913; doi:10.1186/1743-8977-9-40)
Supplement: Additional file 2 — Table S1. Primers used for real-time PCR.Table S2. Selected elements in the exposure atmospheres measured using XRF. [file 1743-8977-9-40-S2.doc]

SUPPLEMENTAL MATERIALS

**Effect of co-exposure to nickel and particulate matter on insulin resistance and mitochondrial dysfunction in a mouse model**

Xiaohua Xu^1^, Xiaoquan Rao^1^, Tse-Yao Wang^1^, Silis Y. Jiang^1^, Zhekang Ying^2^, Cuiqing Liu^2,3^, Aixia Wang^2^, Mianhua Zhong^4^, Jeffrey A. Deiuliis^2^, Andrei Maiseyeu^2^, Sanjay Rajagopalan^2,5^, Morton Lippmann^4^, Lung-Chi Chen^4,*^, Qinghua Sun^1, 2, 5,*^

^1^Division of Environmental Health Sciences

College of Public Health

The Ohio State University

Columbus, Ohio, USA

^2^Davis Heart and Lung Research Institute,

The Ohio State University

Columbus, Ohio, USA

^3^Department of Physiology,

Hangzhou Normal University, Hangzhou, China

^4^Department of Environmental Medicine,

School of Medicine

New York University

Tuxedo, New York, USA

^5^Division of Cardiology

College of Medicine

The Ohio State University

Columbus, Ohio, USA

* Contributed equally.

**Corresponding author:**

Qinghua Sun, M.D., Ph.D.

Division of Environmental Health Sciences

Biomedical Research Tower Room 396

460 West 12^th^ Avenue

Columbus, OH 43210, USA

Phone: (614) 247-1560

Fax: (614) 688-4233

E-mail: [sun.224@osu.edu](mailto:sun.224@osu.edu)

**Supplemental Table S1. Primers used for real-time** PCR

| Primer | Forward oligonucleotides | Reverse oligonucleotides |
| --- | --- | --- |
| *Ucp1* | 5′-ACTGCCACACCTCCAGTCATT-3′ | 5′-CTTTGCCTCACTCAGGATTGG-3′ |
| *Prdm16* | 5′-CAGCACGGTGAAGCCATTC-3′ | 5′-GCGTGCATCCGCTTGTG-3′ |
| *Pgc-1α* | 5′-CCCTGCCATTGTTAAGACC-3′ | 5′-TGCTGCTGTTCCTGTTTTC-3′ |
| *Dio2* | 5′-AAGGCTGCCGAATGTCAACGAATG-3′ | 5′-TGCTGGTTCAGACTCACCTTGGAA-3′ |
| *Cidea* | 5′- ATCACAACTGGCCTGGTTACG-3′ | 5′- TACTACCCGGTGTCCATTTCT-3′ |
| *Elovl3* | 5′- GATGGTTCTGGGCACCATCTT-3′ | 5′- CGTTGTTGTGTGGCATCCTT-3′ |
| *β-actin* | 5′-TGTGATGGTGGGAATGGGTCAGAA-3′ | 5′-TGTGGTGCCAGATCTTCTCCATGT-3′ |

**Supplemental Table S2. Selected elements in the exposure atmospheres measured using XRF**

| ng/m3 |  |  |  |  |  |
| --- | --- | --- | --- | --- | --- |
|  | Ambient | Ni | FA | CAPs+Ni | CAPs |
| S | 578.5 | 286.9 | 25.2 | 6462.2 | 6793.7 |
| Na | 50.7 | 47.3 | -32.8 | 523.9 | 397.6 |
| Si | 34.0 | 55.0 | -33.6 | 473.3 | 500.7 |
| Ni | -0.4* | 440.6 | 0.0 | 467.9 | 0.9 |
| Al | 108.5 | 209.9 | 278.4 | 411.7 | 505.7 |
| Fe | 22.8 | -0.6 | 3.1 | 236.7 | 258.9 |
| K | 19.6 | 10.4 | -5.9 | 229.6 | 244.4 |
| Cl | 13.0 | 36.6 | 3.5 | 211.4 | 173.1 |
| Ca | 19.4 | 45.7 | 11.1 | 180.7 | 162.4 |
| Mg | 14.3 | 76.4 | 50.8 | 113.5 | 183.3 |
| Zn | 7.0 | 13.6 | 5.5 | 66.2 | 67.3 |
| Sn | 8.9 | 66.4 | 49.6 | 46.4 | 48.5 |
| Pb | 7.6 | 11.7 | 10.5 | 32.7 | 39.7 |
| Mn | 1.5 | 20.7 | 1.8 | 28.1 | 7.3 |
| Cu | 4.0 | 13.6 | 7.8 | 23.8 | 19.2 |
| Br | 1.4 | -1.7 | -1.8 | 21.0 | 24.3 |
| Cd | 17.6 | 20.4 | 12.2 | 16.2 | 4.2 |
| Ba | 1.9 | 2.2 | 4.5 | 13.9 | 10.7 |
| Cr | 2.4 | 9.1 | 10.2 | 10.9 | 11.0 |
| Ti | 0.9 | -3.6 | -3.2 | 10.5 | 13.8 |
| Se | 0.4 | 0.2 | 0.2 | 7.1 | 8.5 |
| V | -0.3 | -2.5 | -3.8 | 0.3 | 1.8 |
| As | -3.3 | -12.8 | -11.5 | -8.5 | -8.5 |

*below MDL.
